# Nonrestorative sleep scale: a reliable and valid short form of the traditional Chinese version

**DOI:** 10.1007/s11136-020-02523-4

**Published:** 2020-05-16

**Authors:** S. Li, D. Y. T. Fong, J. Y. H. Wong, K. Wilkinson, C. Shapiro, E. P. H. Choi, B. McPherson, E. Y. Y. Lau, C. L. K. Lam, L. X. Huang, M. S. M. Ip

**Affiliations:** 1 School of Nursing, The University of Hong Kong, 21 Sassoon Road, Hong Kong, China; 2 Department of Psychiatry, University of Toronto, 399 Bathurst Street, Toronto, Canada; 3 Division of Speech and Hearing Sciences, The University of Hong Kong, Pokfulam Road, Hong Kong, China; 4 Department of Psychology, The Education University of Hong Kong, 10 Lo Ping Road, Hong Kong, China; 5 Department of Family Medicine and Primary Care, The University of Hong Kong, 21 Sassoon Road, Hong Kong, China; 6 Department of Mechanical Engineering, The University of Hong Kong, Pokfulam Road, Hong Kong, China; 7 Department of Medicine, The University of Hong Kong, 21 Sassoon Road, Hong Kong, China

**Keywords:** Item response theory, Item selection, Nonrestorative sleep, Optimal test assembly, Reliability, Validity

## Abstract

**Purpose:**

Previous research has suggested the essential unidimensionality of the 12-item traditional Chinese version of the Nonrestorative Sleep Scale (NRSS). This study aimed to develop a short form of the traditional Chinese version of the NRSS without compromising its reliability and validity.

**Methods:**

Data were collected from 2 cross-sectional studies with identical target groups of adults residing in Hong Kong. An iterative Wald test was used to assess differential item functioning by gender. Based on the generalized partial credit model, we first obtained a shortened version such that further shortening would result in substantial sacrifice of test information and standard error of measurement. Another shortened version was obtained by the optimal test assembly (OTA). The two shortened versions were compared for test information, Cronbach’s alpha, and convergent validity.

**Results:**

Data from a total of 404 Chinese adults (60.0% female) who had completed the Chinese NRSS were gathered. All items were invariant by gender. A 6-item version was obtained beyond which the test performance substantially deteriorated, and a 9-item version was obtained by OTA. The 9-item version performed better than the 6-item version in test information and convergent validity. It had discrimination and difficulty indices ranging from 0.44 to 2.23 and − 7.58 to 2.13, respectively, and retained 92% of the test information of the original 12-item version.

**Conclusion:**

The 9-item Chinese NRSS is a reliable and valid tool to measure nonrestorative sleep for epidemiological studies.

**Electronic supplementary material:**

The online version of this article (10.1007/s11136-020-02523-4) contains supplementary material, which is available to authorized users.

## Introduction

Nonrestorative sleep (NRS) refers to the subjective feeling of being restless or un-refreshed even with normal sleep duration [1, 2]. Estimates of the prevalence of NRS may range from 2.4 to 43% in the general adult population across different ethnic groups or using different instruments [3–6], while its prevalence in Hong Kong has been found to be 8.1% [7]. NRS may cause health problems such as fatigue during the day, resulting in decreased daytime function and performance, and increased occupational and nonoccupational accidents [8]. In addition, reduced psychological well-being, mental problems, and chronic disease have also been observed to be strongly associated with NRS [7, 9–11]. NRS sometimes does and sometimes does not coexist with other sleep problems, and hence has been viewed as a discrete treatment target in recent years [12].

In order to properly assess NRS, Wilkinson and Shapiro developed the first appropriate assessment instrument, the Nonrestorative Sleep Scale (NRSS) [13]. The original English version of the NRSS comprised 12 items [13], covering 4 domains: refreshment from sleep, physical/medical symptoms of NRS, daytime functioning, and affective symptoms of NRS. This 12-item NRSS has been translated into traditional Chinese script, with both the original English and traditional Chinese versions demonstrating a valid, reliable 4-factor structure for assessing NRS [13, 14]. Moreover, the traditional Chinese NRSS showed essential unidimensionality in a bifactor analysis [14]. Although the 12-item NRSS is well established, whether it can be shortened, in order to reduce response burden and increase respondent acceptability, has not been explored.

The traditional classical test theory approach assumes constant reliability across all scores, an assumption that is often violated in reality [15]. Moreover, the scale properties obtained on this basis are usually sample dependent, which implies the need to test these properties again in other populations [15]. In contrast, modern item response theory (IRT) focuses on the item level and is sample independent. Moreover, IRT makes it possible to select items that offer more information concerning the measured trait. In this way, a well-performing shortened scale that retains an adequate amount of information along with the precision of the original scale can be obtained [16]. However, the item performance of the NRSS has not been examined using IRT.

Therefore, this study aimed to use develop a short version of the Chinese NRSS to facilitate the quick and efficient assessment of NRS.

## Methods

### Participants

Individuals who were 18 years old or above and were able to read traditional Chinese or communicate in Cantonese were recruited. Those who were not willing to participate or had difficulty understanding the study procedures were excluded. In addition, people who were taking medication for sleep disorders or had psychiatric illnesses were excluded. To conduct a generalized partial credit model (GPCM) for the analysis of polytomous responses, a sample size of at least 250 respondents is acceptable [17]; hence, a sample of 250 respondents or more was targeted, and ultimately a total of 404 subjects were recruited across 2 studies.

### Procedures

A total of 404 people who had completed the 12-item Chinese NRSS were gathered from 2 studies that had the same eligibility criteria. The first was a cross-sectional study that recruited 120 participants by telephone and home visit between September 2016 and July 2017 [14]. The second was a household survey that recruited 284 participants between May 2018 and March 2019. Potential participants were identified from a list of household addresses maintained by the Hong Kong Census and Statistics Department. The records of household addresses were grouped into quarters, and a random sample of the quarters was obtained by systematic sampling design with fixed sampling intervals and nonrepetitive random numbers; all households residing in the selected quarters were covered in the survey. In both studies, when there was more than one eligible person living in the household, the person closest to their next birthday was recruited. Ethics approval for both studies was obtained from the Institutional Review Board of the University of Hong Kong/Hospital Authority Hong Kong West Cluster (Ref nos. UW16-326 and UW 17-011).

### Measurements

#### The nonrestorative sleep scale (NRSS)

The 12-item NRSS has 6 items that require reverse coding. Moreover, 10 items require responses on a 1–10 scale with adjacent responses scored as 1–5, that is, responses 1 and 2 scored as 1, responses 3 and 4 scored as 2, etc. The other 2 items require responses scored on a 1–5 scale. A higher total scale score corresponds to less NRS [13].

#### The pittsburgh sleep quality index (PSQI)

The PSQI was developed to evaluate sleep quality during the past month [18]. The items are grouped into 7 components, including subjective sleep quality, sleep latency, sleep duration, habitual sleep efficiency, sleep disturbance, use of sleeping medications, and daytime dysfunction, with each rated on a 0–3 scale [18]. The total score ranges from 0 to 21, with a higher score indicating poorer sleep quality [19]. Previous studies used PSQI to test the convergent validity with NRSS [13, 14].

### Statistical analysis

The scale scores were summarized with descriptive statistics. NRSS item reduction was done by examining item characteristics using IRT analysis.

We first assessed the two assumptions of IRT: unidimensionality and local independence [20]. Unidimensionality for the 12-item NRSS was previously shown in a bifactor model [14]. Nevertheless, we retested this assumption in our sample using minimum residual factoring of the polychoric correlation matrix exploratory factor analysis (EFA) [21]; specifically, we assessed Kaiser–Meyer–Olkin (KMO) and Bartlett’s test of sphericity values to confirm the appropriateness of the EFA [22]. Then, the number of factors was identified by assessing the scree plot [23]. Unidimensionality was accepted if the first factor explained more than 20–40% of variance [24, 25] and the ratio of the eigenvalues of the first to the second unrotated factor was greater than 3 [20]. Local independence refers to independence among responses across items conditioned on the corresponding latent trait [26]; its presence was accepted if the residual correlations for the items were smaller than 0.25 [27]. After confirming unidimensionality and local independence, we fitted a GPCM.

Under the GPCM, we obtained the discrimination parameter (*a*_*i*_) and the difficulty parameters (*b*_*i*_) for each item. A higher discrimination parameter value indicates greater ability of the corresponding item to differentiate respondents at different trait levels. In this study, ability refers to the NRS level. The discrimination parameter often ranges from 0.5 to 2.5, and items with values smaller than 0.4 are recommended for removal [28]. As for the number of difficulty parameters, it corresponds to the number of response categories minus 1. Under the GPCM, the difficulty parameter refers to the latent trait level where the probabilities of endorsing the two adjacent response categories are the same [29]. Item characteristic curves (ICCs) were also obtained, with the curve steepness reflecting the discrimination level: greater steepness demonstrated greater discrimination ability [30].

To examine differential item functioning (DIF) by gender, that is, to determine whether there were items that were responded differently by male and female participants even when they had the same trait level [31], similarity of slopes and intercepts by gender was tested using an iterative Wald approach, with a significant p-value indicating DIF [32]. Specifically, the Wald-2 approach was used to identify the anchor items, that is, items showing invariance across groups. To better control type 1 error rate, we also adopted the MaxA5 method, which uses 5 items with the largest discrimination parameters as the anchor items [33], before the Wald-1 approach was iteratively used to test for DIF items [32]. The female group was set as the reference group and the male group was set as the focal group.

In addition, we also obtained item information, test information and average standard error (SE). Test information, which is the sum of the item information for all items, provides evidence on how accurately the test estimates a latent trait over the entire range of trait levels. The more information at a particular trait level provided by the test, the higher the precision of ability estimation, and the higher the reliability [34]. Test information was obtained over the entire range of latent trait levels (*θ*), as well as over the common range of (− 3, 3) used to avoid potentially inflated information due to the presence of extremely able/unable participants [35]. The SE refers to the standard error of latent trait estimates, which indicates the amount of information unexplained by the items being considered. It is independent of the distribution of scores in the obtained sample [36]. It was taken as the average of SE at each latent trait level in the study.

We tried two item selection approaches that had been adopted in the literature. First, the item reduction process was initiated by removing, first, items with discrimination < 0.4 and then items that showed DIF by gender. The process continued with the assessment of test information and SE of measurement. Specifically, first, the item that carried the lowest item information was removed, and test information and SE were assessed. Then, the item that carried the next-lowest item information was removed and test information and SE again assessed. We continued the removal of items until there was a relatively substantial reduction in test information and increase in SE. Finally, this shortened NRSS was tested again for item discrimination and difficulty using IRT.

Second, optimal test assembly (OTA) was adopted, after excluding the items showing DIF. For each fixed number of items between 3 and 12, the set of NRSS items that maximized the total test information over five anchor points (*θ*: − 3, − 1, 0, 1, 3) based on GPCM was first obtained using the branch-and-bound algorithm [37]. Then, the shortened version was taken as the smallest set of items that satisfied three criteria: (1) the correlation between the factor scores (as well as summed scores) of the shortened version and those of the 12-item version should be at least 0.95; (2) the convergent validity correlation between the factor scores (as well as summed scores) and PSQI should be within a tolerance of 0.05 when compared with that of the 12-item version; and (3) the Cronbach’s alpha should be at least 95% of that of the 12-item version.

The obtained shortened versions were compared with the original 12-item version in terms of test information, Cronbach’s alpha, and convergent validity.

The EFA was conducted with the package “psych” [38], while DIF was tested with the package “mirt” [39]. The R package “ltm” was used to run the OTA procedure [40]. The OTA was procedure was implemented through the package “lpSolveAPI” [41]. All of the packages were run in RStudio 1.1.383. SEs for each latent trait level were obtained in IRTPRO (4.2 Student version).

## Results

### Participant demographic characteristics

The combined sample had 162 male and 242 female respondents. Their average age was 45 years (standard deviation: 17; range: 18–88). In all, 38 participants (9.4%) had primary education or below, 213 (52.7%) had secondary education, and 151 (37.4%) had a bachelor’s degree or above. There were 238 (58.9%) workers, 60 (14.9%) homemakers, 31 (7.7%) students, 68 (16.8%) retired participants, and 6 (1.5%) job-seeking participants. All participants completed the 12-item NRSS without any missing values.

### Checking IRT assumptions for the full NRSS

The KMO statistic was 0.86 and Bartlett’s test was statistically significant (*p* < 0.001). The EFA identified one factor, with the eigenvalue of the first factor (4.6) substantially larger than those of the next 2 factors (1.3 and 0.7, respectively). The first rotated factor explained 38% of total variance, much higher than the second factor, which explained only 11%; therefore, the 12-item test could be regarded as essentially unidimensional. Moreover, the residual correlations among the 12 items ranged from 0.21 to 0.22, all smaller than 0.25; hence, the local independence assumption was also met.

### NRSS item properties and selection

Table 1 shows the values of the discrimination and difficulty parameters for the 12 items of NRSS. Discrimination for the 12 items ranged from 0.33 to 2.10. Items with the highest 5 discrimination parameters were Q10 (*a* = 2.10), Q1 (*a* = 1.74), Q2 (*a* = 1.39), Q9 (*a* = 1.36), and Q3 (*a* = 1.24). Appendix 1 in shows the ICCs of all 12 NRSS items. Table 1 also shows the total information of each item which lay between 1.29 and 8.41. Item 5 was removed due to the small discrimination parameter.

**Table 1:** The discrimination, difficulty, and item information of the 12 Chinese NRSS items

Items		Difficulty parameters (*b*_*ij*_)				Item information
	*a*_*i*_	*b*_*i1*_	*b*_*i2*_	*b*_*i3*_	*b*_*i4*_	
Q1: …rate the quality of your sleep	1.74	− 2.12	− 1.20	− 0.12	2.14	6.94
Q2: …sleep is restoring or refreshing?	1.39	− 3.21	− 1.70	− 0.50	1.55	5.57
Q3: …felt rested if you’ve slept for your usual amount of time?	1.24	− 2.08	− 0.84	− 0.27	1.76	4.97
Q4: …had physical sensations or unusual feelings?	0.43	− 5.26	− 1.83	0.03	− 0.54	1.66
Q5: …one or more of the following: headaches, body pain, numbness, or tingling…?	0.33	− 0.72	− 4.68	− 1.58	− 2.87	1.29
Q6: physical or medical problems are dragging you down?	0.65	− 4.94	− 1.41	0.73	− 0.61	2.56
Q7: … have a sense of panic, or physical symptoms of panic…?	0.53	− 6.42	− 1.67	− 0.81	− 1.74	2.06
Q8: …memory and concentration…?	1.12	− 2.07	− 1.65	− 0.42	2.20	4.49
Q9: …level of daytime energy?	1.36	− 2.50	− 1.73	− 0.44	2.14	5.45
Q10: …alert during the daytime?	2.10	− 2.20	− 1.77	− 0.74	1.46	8.41
Q11: …depressed or down if you didn’t sleep well…?	0.40	− 5.64	− 1.29	0.37	− 0.18	1.54
Q12: …irritable or “gotten the blahs” if you didn’t sleep well	0.98	− 2.50	− 2.88	− 1.07	− 0.09	3.90

*a*_*i*_ the discrimination parameter, i.e., the ability of an item to discriminate different levels of nonrestorative sleep, *b*_*ij*_ difficulty parameter, represents the latent trait level of choosing response *j* instead of *j*−1 of item *i*

Using the iterative Wald test approach, the Wald-2 test (all-others-as-anchors model) revealed DIF by gender only in Q8. However, after setting the five items with the largest discrimination parameters as anchor items in Wald-1 test, Q8 had a *p *value of 0.061 and thus was concluded to be invariant by gender.

Appendix 2 graphically shows changes in test information and SE as more items were removed. When Q5, Q11, Q4, Q7, Q6, and Q12 were removed, the additionally removed Q8 resulted in greater increase of SE and decrease of test information when compared with the original 12-item scale. Therefore, we retained 6 items—Q1, Q2, Q3, Q8, Q9, and Q10. The OTA procedure and decision rules removed Q4, Q5, and Q11, which had the smallest discrimination parameters and item information, resulting in a 9-item scale—Q1, Q2, Q3, Q6, Q7, Q8, Q9, Q10, and Q12. The 9-item scale had a Cronbach’s alpha value of 0.835. The correlation between the summed scores of the 9-item and 12-item versions was 0.960, while that between the factor scores was 0.995. The correlations between the PSQI and the 9-item summed and factor scores were 0.975 and 0.966, respectively.

### Testing the shortened traditional Chinese NRSS scales

IRT analysis on the 6-item Chinese NRSS showed that all items had discrimination parameters ranging from 1.26 to 2.22, while difficulty parameters ranged from − 3.13 to 2.13. The discrimination parameters and difficulty parameters for the 9-item scale ranged from 0.44 to 2.23, and from − 7.58 to 2.13, respectively.

**Fig. 1:**
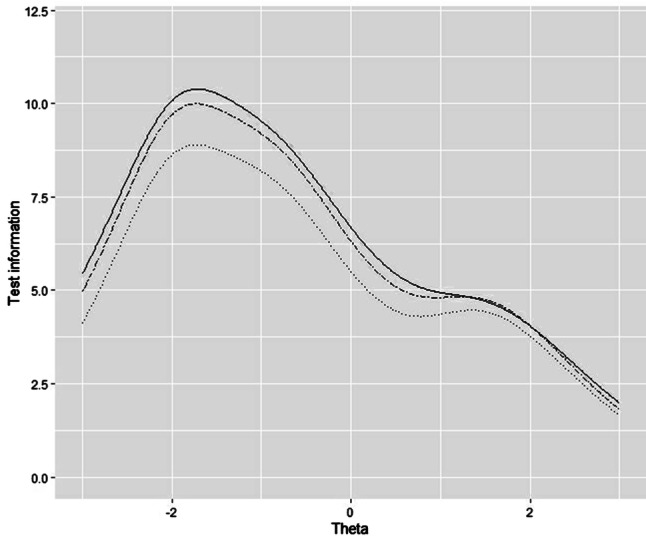
Test information curves of the original 12-item NRSS (solid curve), the 9-item NRSS (longdash curve), and 6-item NRSS (dotted curve)

Figure 1 shows the test information for the original 12-item, the 6-item, and the 9-item NRSS versions between NRS level (*θ*) range of (− 3, 3). The test information of the 9-item version closely resembled that of the original 12-item version. Table 2 compares the test information, Cronbach’s alpha, and convergent validity with PSQI of the different versions. The 6-item and the 9-item versions, respectively, kept 78% and 92% of the test information over the entire *θ* range of the original 12-item scale, and kept 84% and 95% between *θ* (− 3, 3). The Cronbach’s alpha was quite similar across all versions. The 9-item version had higher convergent validity and test information, and kept at least one item for each of the four domains in the 12-item version. Therefore, the 9-item version performed better than the 6-item version.

**Table 2:** Comparison on test information, Cronbach’s alpha, and convergent validity of different versions of NRSS

	12 items	9 items	6 items
Test information^a^	48.87	45.07	38.28
Test information (− 3, 3)^b^	40.09	38.27	33.64
Cronbach’s alpha	0.834	0.835	0.843
Convergent validity	− 0.617	− 0.613	− 0.547

^a^Test information over the entire latent trait level

^b^Test information over the latent trait level (*θ*) of (− 3, 3)

## Discussion

This study has made the first attempt to develop a short form NRSS for assessing NRS. Using the IRT and OTA methodology, we obtained a 9-item scale that kept at least one item from each of the 4 hypothesized domains of the NRSS. All the retained items had relatively high discriminative power, and the shortened scale had test information closely resembling that of the original 12-item scale.

Higher discrimination of an item means that the probability of choosing a particular response for that item will increase more rapidly as the ability level of a respondent increases [42]. Baker proposed an interpretation of discrimination values as follows: *0* = nondiscriminative; 0.01–0.34 = very low; 0.35–0.64 = low; 0.65–1.34 = moderate; 1.35–1.69 = high; > 1.70 = very high [30]. The discrimination parameters for the 9 retained items ranged from 0.44 to 2.23, corresponding to low-to-high discrimination for assessment of NRS. However, we kept the item with the discrimination of 0.44 as a cut off value of 0.4 has been suggested.

Using the Wald-2 test, gender-related DIF was detected on item Q8, which asked “How is your memory and concentration during the daytime?,” but no DIF was detected in the second stage by Wald-1 test. The Wald-2 is known to be more likely to inflate Type I error rate [43]. This study adopted the iterative Wald test approach, which was shown, in simulation study, to outperform the Wald-1 and Wald-2 tests when they were used individually, in that it had lower Type I and Type II error rates [32, 33]. Nevertheless, since the *p *value of 0.061 was marginal, more data and studies are worth pursuing.

Based on OTA with GPCM, items Q5, Q11, and Q4 were removed. These three items carried low information and were all negatively worded. Previous study has indicated that question phrasing methods may impact the performance of a questionnaire with Chinese respondents [44]. In particular, Q5 asked about symptoms such as “headaches, body pain, numbness, tingling, nausea, racing heart/palpitations, sore throat, frequent cough”; however, the extent to which such specific symptoms are associated with NRS has not been well determined, and more studies are necessary to confirm such associations, if any. Q11 asked “Do you feel depressed or down if you didn’t sleep well the night before?.” Depression or depressed feeling may take an extended time to develop and would be unlikely to develop merely if one had not slept well recently, ceteris paribus. Q4 asked “Have you had physical sensations or unusual feelings in your body that you couldn’t identify?.” Its low information value is indeed consistent with what we observed in our post-test debriefing, when some people stated they were unsure how one could have physical sensations or unusual feelings that could not be identified.

The 9-item version retained 92% and 95% of the test information of the original 12-item version over the entire NRS level range and the range of (− 3, 3), respectively. Their test information functions were very close to each other and were the highest over around the same range of the latent trait. These findings demonstrate that adoption of the shortened 9-item version would not substantially sacrifice precision or reliability.

Recently, OTA has demonstrated value in developing short forms of PROs [45, 46]. It offers an appealing solution for determining a replicable and reproducible short form. Nevertheless, despite our efforts in conducting a rigorous study, there are several limitations worth noting and also worth addressing in future work. First, although our study size was adequate for typical IRT analysis, a larger population-based study would be desirable to establish Chinese NRSS short form norms that can facilitate the interpretation of the tool. Second, DIF analysis for other participant groups, such as language groups, should also be done, for cross-cultural comparison. Third, the multidimensional IRT has been made available to allow IRT to be conducted on multidimensional scales. Since the traditional Chinese NRSS is essentially unidimensional, we did not pursue multidimensional IRT. Lastly, the reliability and validity of the 9-item scale would preferably be further examined in an independent sample.

Conclusively, nevertheless, the 9-item traditional Chinese NRSS is a reliable and valid tool for assessing NRS. The short form allows more efficient assessment by healthcare professionals, researchers, and the public.

## Electronic supplementary material

Below is the link to the electronic supplementary material.

Supplementary material 1 (TIF 416 kb)

Supplementary material 2 (TIF 193 kb)
