# Supplementary figures and images for: Nonrestorative sleep scale: a reliable and valid short form of the traditional Chinese version
Source: Qual Life Res. 2020 May 16;29(9):2585–92. doi: 10.1007/s11136-020-02523-4 (PMC7434790; doi:10.1007/s11136-020-02523-4)

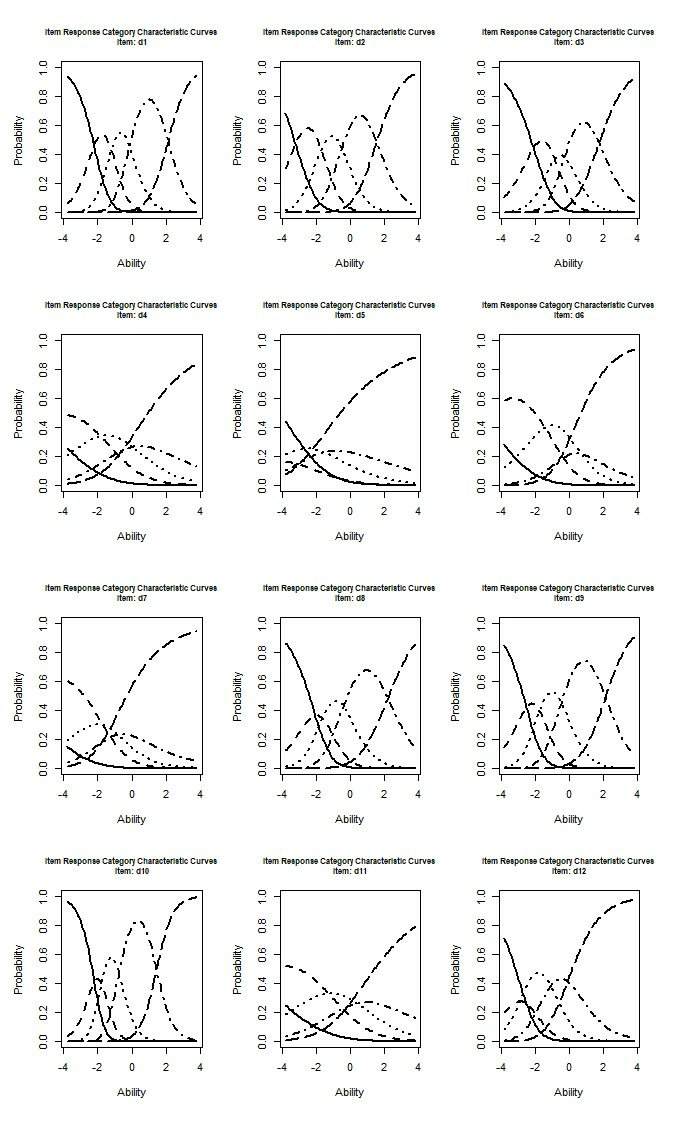

Supplement: Supplementary file 1 — Supplementary material 1 (TIF 416 kb) [file 11136_2020_2523_MOESM1_ESM.tif]

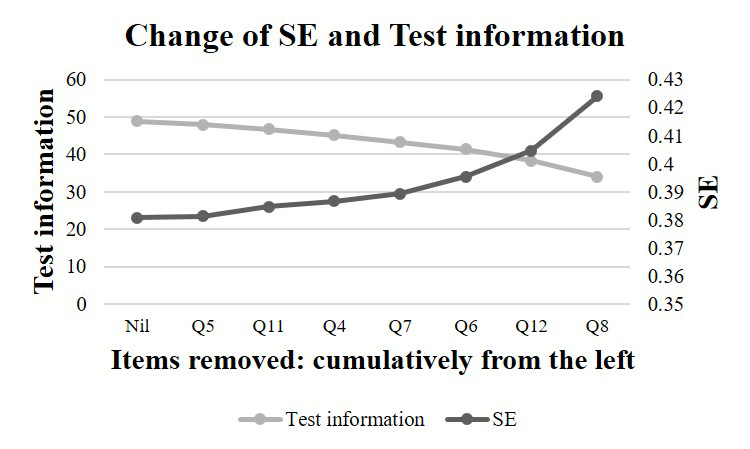

Supplement: Supplementary file 2 — Supplementary material 2 (TIF 193 kb) [file 11136_2020_2523_MOESM2_ESM.tif]
